# Influence of the Presence of Tissue Expanders on Energy Deposition for Post-Mastectomy Radiotherapy

**DOI:** 10.1371/journal.pone.0055430

**Published:** 2013-02-06

**Authors:** Débora M. Trombetta, Simone C. Cardoso, Alessandro Facure, Ademir X. da Silva, Luiz Antonio R. da Rosa

**Affiliations:** 1 Programa de Engenharia Nuclear/Instituto Alberto Luiz Coimbra de Pós-graduação e Pesquisa de Engenharia (COPPE) - Universidade Federal do Rio de Janeiro (UFRJ), Rio de Janeiro, RJ, Brazil; 2 Laboratório de Física da Radiação Gama/Instituto de Física (IF) - Universidade Federal do Rio de Janeiro (UFRJ), Rio de Janeiro, RJ, Brazil; 3 Comissão Nacional de Energia Nuclear (CNEN) - Rio de Janeiro, RJ, Brazil; 4 Instituto de Radioproteção e Dosimetria (IRD) - Comissão Nacional de Energia Nuclear (CNEN),Rio de Janeiro, RJ, Brazil; Dartmouth, United States of America

## Abstract

An increasing number of studies have shown that post-mastectomy radiotherapy presents benefits associated with the patients survival and a significant fraction of the treated patients makes use of tissue expanders for breast reconstruction. Some models of tissue expanders have a magnetic disk on their surface that constitutes heterogeneity in the radiation field, which can affect the dose distribution during the radiotherapy treatment. In this study, the influence of a metallic heterogeneity positioned in a breast tissue expander was evaluated by means of Monte Carlo simulations using the MCNPX code and using Eclipse treatment planning system. Deposited energy values were calculated in structures which have clinical importance for the treatment. Additionally, the effect in the absorbed energy due to backscattering and attenuation of the incident beam caused by the heterogeneity, as well as due to the expansion of the prosthesis, was evaluated in target structures for a 6 MV photon beam by simulations. The dose distributions for a breast treatment were calculated using a convolution/superposition algorithm from the Eclipse treatment planning system. When compared with the smallest breast expander volume, underdosage of 7% was found for the largest volume of breast implant, in the case of frontal irradiation of the chest wall, by Monte Carlo simulations. No significant changes were found in dose distributions for the presence of the heterogeneity during the treatment planning of irradiation with an opposed pair of beams. Even considering the limitation of the treatment planning system, the results obtained with its use confirm those ones found by Monte Carlo simulations for a tangent beam irradiation. The presence of a heterogeneity didńt alters the dose distributions on treatment structures. The underdosage of 7% observed with Monte Carlo simulations were found for irradiation at 0°, not used frequently in a clinical routine.

## Introduction

Post-mastectomy radiotherapy is employed with the purpose of destroying cancer cells that remain after surgery. Most women, who are submitted to mastectomy surgery, undergo breast reconstruction after that procedure and, when great resections are made without sufficient breast tissue to achieve the reconstruction, distant flaps or tissue expanders are used.

Tissue expanders with incorporated magnetic valves are widely used in post-mastectomy breast reconstruction. The elastic capacity of natural tissues to be extended without losing vascularization and innervations is the basic concept applied. The tissue expanders are made of a membrane composed by silicone elastomer, a chemically and mechanically resistant material. The tissue expanders have a magnetic valve located inside the membrane that has to be periodically filled with a saline solution until the desired expansion is reached. This valve is equipped with a magnetic disk which allows the determination of its location inside the patients body.

The immediate breast reconstruction helps to reduce the patient’s psychological distress due to an abnormal and asymmetrical body [1] and breast reconstruction using tissue expanders offers excellent symmetry of the reconstructed breast [2]. In addition, tissue expanders give a more natural appearance, with the same color and texture of the skin. Furthermore, the sensibility of the reconstructed breast skin doesn’t change. The expander is placed during the mastectomy surgery and, depending on the desired volume; it can remain within the patients body for up to 8 weeks. Compared to other alternatives, the relative simplicity, low morbidity and fast recovery of patients make reconstruction with tissue expanders an attractive option for patients and surgeons.

Many clinical trials have demonstrated the benefits of performing the post-mastectomy radiotherapy, mainly for high risk breast cancer patients [3]–[5]. Radiotherapy treatment is usually started 4–8 weeks after the mastectomy surgery, thus several patients are using the tissue expander when they are submitted to radiotherapy. The combination of radiotherapy treatment with the use of implants has been studied and most of studies present a high rate of complications when this association occurs [6]–[11]. Nava et al [12] have observed the effects of radiation on temporary expanders and permanent implants in a range of five years, considering a population of 257 patients subdivided in groups including a group of non-irradiated patients. They estimated that failure in the breast reconstruction was significantly higher for patients submitted to radiotherapy with tissue expanders implants. They estimated a 40% of unsuccessful reconstruction with the combination of tissue expander plus radiotherapy, compared with 6.4% for reconstruction with permanent implant plus radiotherapy, and 2.3% for the control group, which did not receive any therapy with radiation, suggesting that the irradiation treatment should be delivered in the last step of reconstruction when permanent implants have already been performed.

Moni et al (2004) performed experimental measurements around the magnetic valve of a McGhan (Inamed/Allergan) tissue expander using films and thermoluminescent dosimeters (TLD) for a 6 MV photon beam, for two beam incidences (parallel and perpendicular to the artifact). The results showed a decrease in the dose measured directly under the metallic port, around 25% in a region of 1.7 to 3.7 cm away from the artifact.

Damast et al (2006) investigated the effect of a McGhan Style 133 (Inamed/Allergan) tissue expander in a radiotherapy treatment using films and TL dosimeters, in a water phantom and *in vivo,* using 6 and 15 MV photon beams at incidences perpendicular and parallel to the heterogeneity. Results showed 22 and 16% of attenuation at 2 cm away from the magnetic valve for the 6 and 15 MV beams, respectively, in the parallel orientation. Chatzigiannis et al (2005) performed Monte Carlo simulations using CT images of a patient implanted with a McGhan Style 133 (Inamed/Allergan) tissue expander. The magnet of the valve was simulated as being composed by Neodymium-iron-boron. Attenuation of 6–13% was found through all the area in the shadow of the magnetic valve and a dose enhancement around 10% was found near the metallic structure.

The most extreme result was found by Thompson and Morgan^16^. They described a 11% dose enhancement in a region of 5 mm around the valve and a reduction of 25% at 5 cm away from the magnetic valve, considering the same tissue expander in both cases. They obtained the results making use of diodes, with a 6 MV photon beam, considering two irradiation orientations, parallel and perpendicular to the implant central axis.

All the previous studies [13]–[16] were developed using only one type of tissue expander (McGhan Inamed/Allergan). The expander volume used was not described and their variation during the Radiotherapy treatment was not taken into account by any of the studies aforementioned.

The present work aims to evaluate the influence of geometry and composition of the magnetic disk and how the expander volume changes could affect the deposited energy and the planning of breast radiotherapy treatment. To achieve this purpose, two approaches were considered. First, Monte Carlo simulations using a breast phantom, considering internal structures similar to the real ones, assessing the energy deposition in relevant structures due to a treatment with a 6 MV photon beam. Following, dose distributions were calculated using a convolution/superposition algorithm from the Eclipse treatment planning system, based on computational tomography (CT) images of an anatomical phantom, to evaluate how the clinical treatment planning was affected by the presence of the magnetic heterogeneity and the increase in the expander volume.

## Methods

### Simulation Code and Irradiation Parameters

The code used for Monte Carlo simulations was the MCNPX [17]. MCNP is a well-known general-purpose Monte Carlo N-Particle code that can be used for neutron, photon, electron or coupled neutron/photon/electron transport within an arbitrary three-dimensional configuration of materials in geometric cells; relating the created geometries with various classes of materials. A variety of sources can be defined by the user, from where electron, photon and neutron emission is simulated, with probability distributions for energy and direction defined by the user. Subsequently, interactions are simulated according to the type of particle and material properties, as well as the production of secondary particles, which can be assessed for both particle fluency and energy deposition. The discrete cross section data used by this code is part of the Evaluated Nuclear Data File (ENDF) and Evaluated Nuclear Data Library (ENDL).

A conic source was simulated with the MCNPX [17], at a distance of 100 cm from the entrance surface of the beam, emitting a divergent 6 MV photon beam. The photons were confined to a cone whose half-angle brought up a circle field shape with radius R equivalent to a square field with side L = 12 cm.

The 6 MV spectrum used in the simulations was extracted from the work of Daryoush and Rogers [18], who did wide-ranging simulations of nine beams from three major medical linear accelerators manufacturers, from 4 to 25 MV equipments. The calculated and measured depth-dose data agree within 1% (local dose), at all depths past depth of maximum dose.

The MCNP *f8 tally was used to calculate the energy deposited in the desired cells, and the number of stories simulated was 10^6^, ensuring that the estimated relative error was always less than 0.3%.

### Magnetic Valve Model

In this study three different magnetic valves used in tissue expanders were considered. These magnetic valves are manufactured by two silicone prosthesis companies: INAMED/Allergan and SILIMED. The last one is the unique company that manufactures tissue expanders in Latin America.

**Figure 1:**
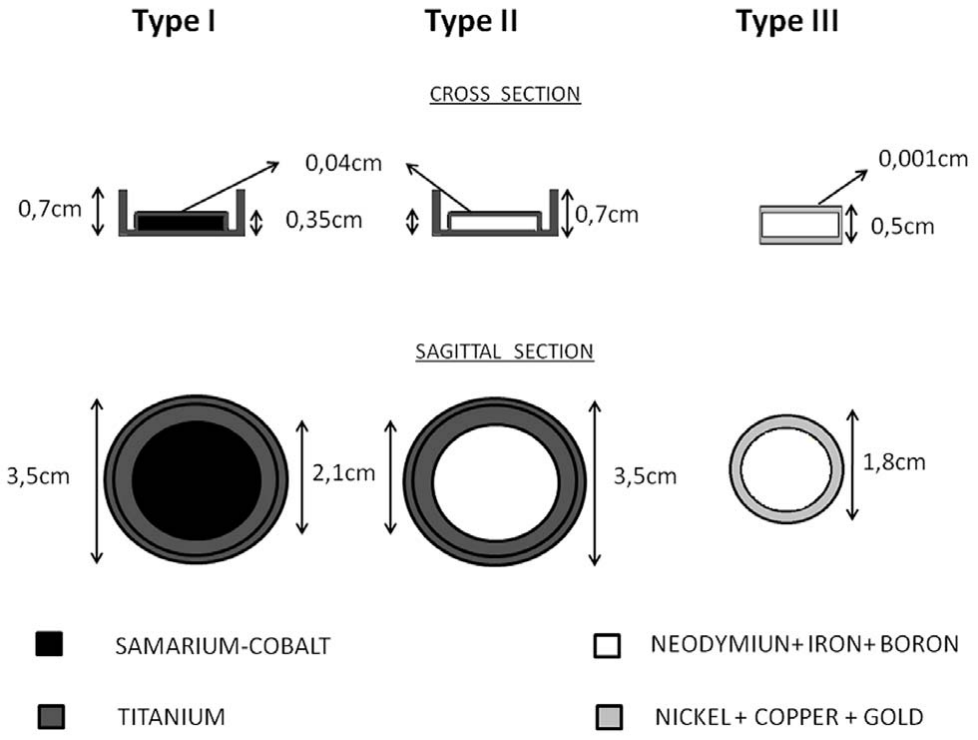
Materials and dimensions for the three types of magnetic valves. Type I and II are manufactured by INAMED/Allergan and type III by SILIMED. The valves are presented in transversal and sagittal views.

Magnetic valves are composed, in general, of a magnetic disk wrapped within an inert material and the most common rare-earth magnets used are Neodymium and Samarium-Cobalt. Three types of valves were simulated with geometries and compositions described in accordance with their manufacturers. Differences in the geometry for the three magnetic valves are shown in the Fig. 1.

**Figure 2:**
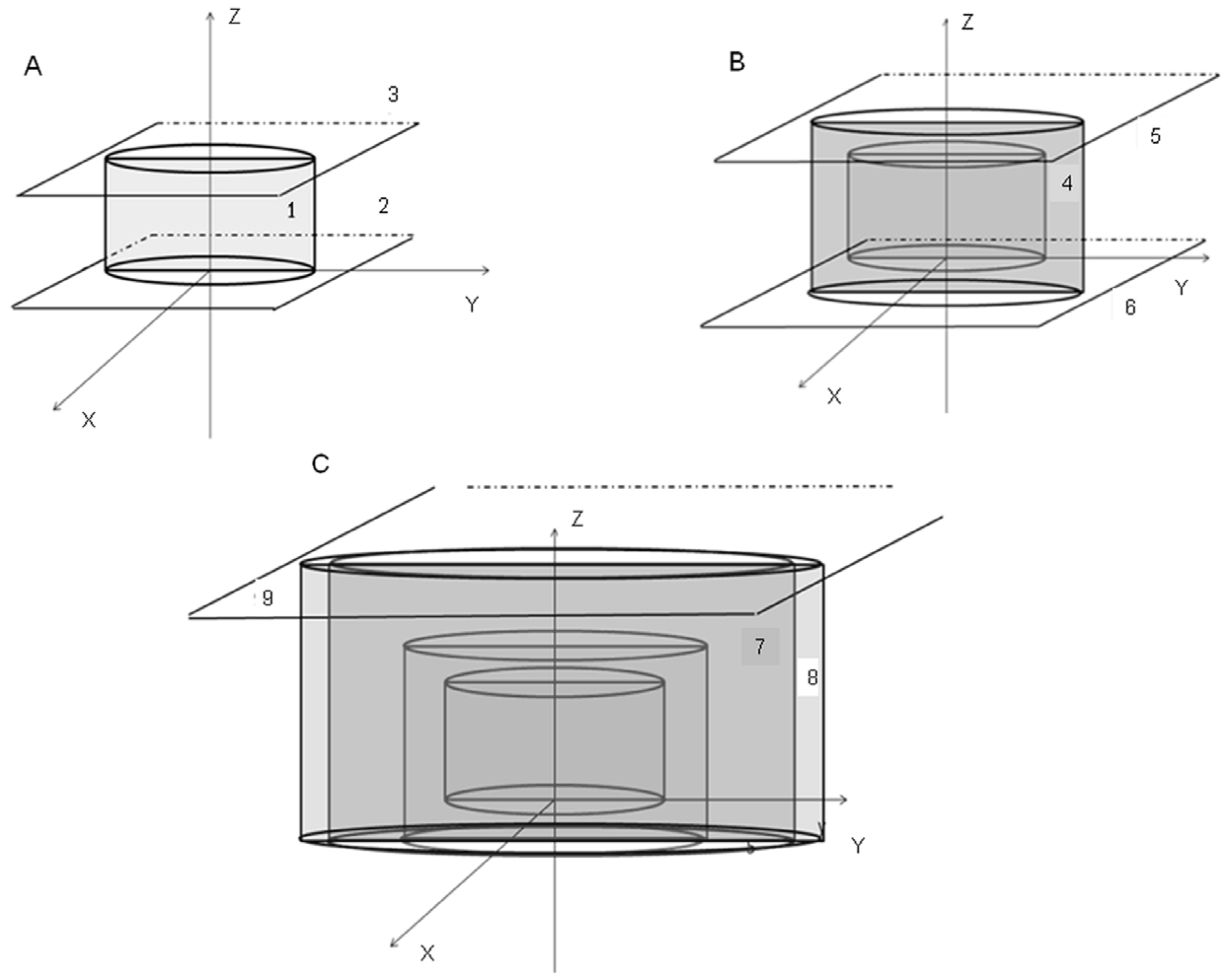
Illustration of the computational simulation of the magnetic valves. They were performed with the intersection between cylinders and planes with MCNPX code. A) Cylinder 1, planes 2 and 3 generate the magnetic disk; B) Cylinder 4, planes 5 and 6 generate the metal capsule; C) Cylinders 7, 8 and planes 6, 9 generate the external cup-shape structure that contains the magnetic disk in the expanders type I and II.

In order to simulate these heterogeneity models, an intersection between geometric figures of cylinders and planes were done (Fig. 2).

The validation of the magnetic heterogeneity’s geometry was performed in a previous study [19], where calculations for the beam attenuation due to the presence of the magnetic valve inside the expander were obtained by Monte Carlo simulations using MCNP (Monte Carlo N-particle) code [17], for a 6 MV photon beam. The simulated attenuation values were compared with experimental measurements done by Damast el al (2006). An excellent agreement, with maximum difference of 3%, was found between the results, which validated the simulated geometry for the magnetic valve.

### Computational Breast Phantom

**Figure 3:**
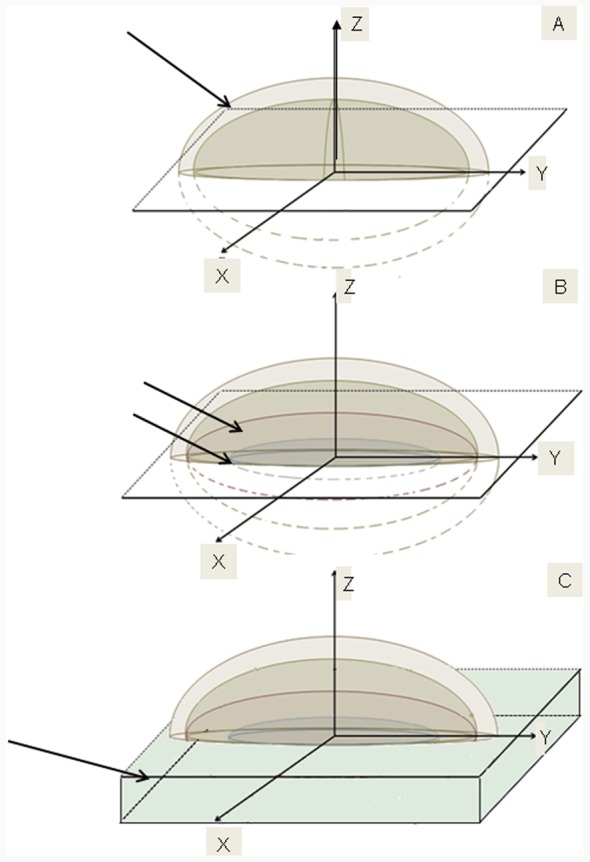
Breast simulator computationally. It was constructed by intersection between concentric ellipsoids and planes. A: Pectoral muscle and skin; B: Chest wall and Lung; C:Water block.

A breast computational phantom was simulated using direct measurements of the distances between the existing structures, inside and near the breast, from de-identified tomographic images. The tomographic images were obtained from patient records at the Brazilian Cancer National Institute. All data were analyzed anonymously and no individually identifiable information was used in this study. The tomographies showed in this paper are of the Alderson Rando anthropomorphic phantom with the breast built for this work. The unique information used from patient is geometric. As the pectoral muscle and skin have similar density and mass attenuation (ρ_skin_ = 1.09 g/cm^3^, μ_1MeV(skin)_ = 0.071 cm^2^/g and ρ_muscle_ = 1.05 g/cm^3^, μ_1MeV (muscle)_ = 0.070 cm^2^/g), these two structures were considered as a single one with 1.2 cm thickness, formed by the intersection between concentric ellipsoids and a plane, whose cross section have a diameter of 18 cm (Fig. 3–A). The chest wall, composed of ribs bones and muscles, was represented below the pectoral muscle by another concentric half ellipsoid, adding a thickness of 1 cm (Fig. 3–B). The influence of bone ribs in the absorbed energy was assessed through calculations. Three different geometries were simulated, first considering only muscle tissue (1 cm of thickness), then muscle (0.4 cm) and bone tissue (0.6 cm), actual geometry, and finally only bone tissue (1.0 cm), taking into account the presence and the absence of the bone structure.

As an almost 2 cm thick portion of lung lies within the treatment field, this organ was represented in the computational phantom by a circular cross section with a diameter of 11 cm, composed by lung tissue (Fig. 3–B). Just below the phantom, a water block was simulated in order to avoid border effects between the interfaces of phantom/air (Fig. 3–C). The density used in the each breast simulator model component was obtained from ICRU 46 Publication [20].

**Figure 4:**
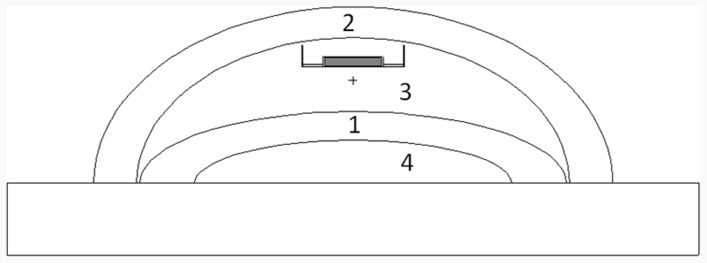
Breast simulator with the representative structures. 1-chest wall, 2-breast tissue and skin, 3-tissue expander and 4-lung.

The expander was simulated between the representative structures of the chest wall and pectoral muscles. Its volume was varied from 100 to 600 ml, in increments of 60 ml. As shown in the Fig. 4, the magnetic disk was positioned on the top of the structure which represents the expander. This is the most common and simple position where it is placed, but there are also expanders on which the disk is shifted to one side in order to generate a different shape when within the patient’s breast.

### Calculation of Absorbed Energy Using Monte Carlo Simulations

**Figure 5:**
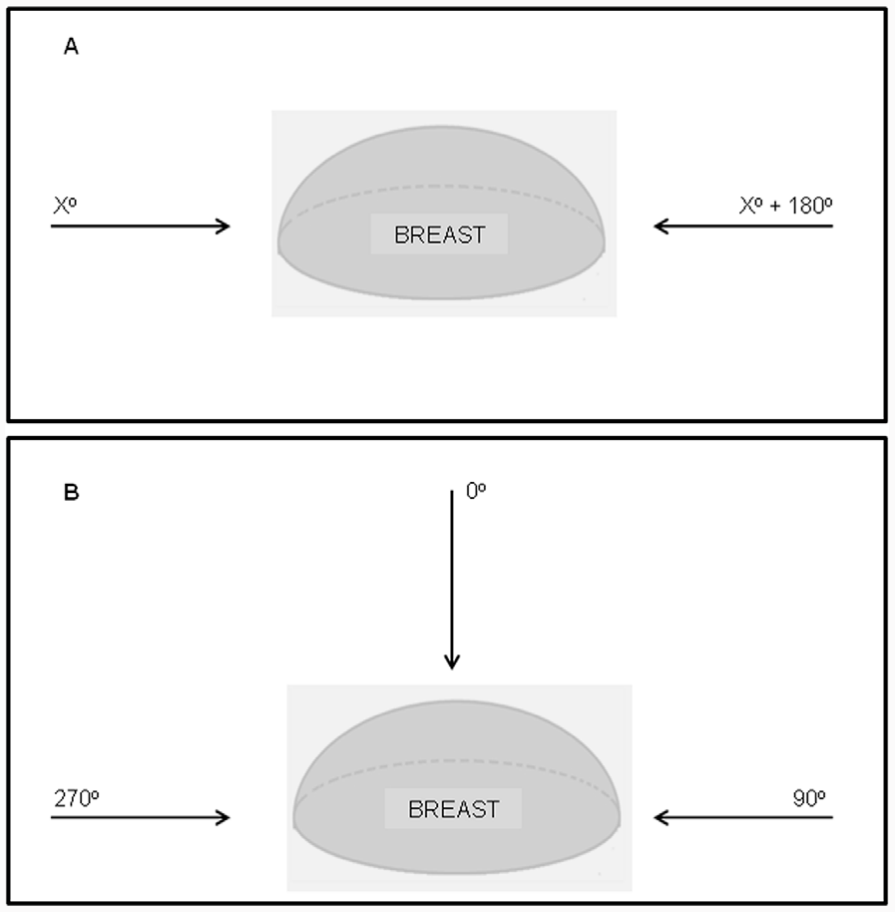
Positions representing the usual technique of breast irradiation. The first one is the real case and the second one the setup simulated in this work.

In post-mastectomy radiotherapy treatment, it is common the use of two opposed tangential fields for the whole breast and chest wall irradiation, as depicted in (Fig. 5–A). However, as most studies available in the literature evaluate a different geometry of irradiation, for comparison, the breast phantom was irradiated with the source in three extreme positions: 0°, 90° and 270°. These irradiation positions are illustrated in the Fig. 5–B. In order to evaluate the effect produced by the magnet in the radiation field, computational breast phantom simulations performed without the magnetic disk (heterogeneity) were also performed.

Radiotherapy breast treatment aims to irradiate remaining tissues and sometimes the chest wall. Therefore, the deposited doses in the structures above the expander (skin) and below it (chest wall) were calculated, allowing for the assessment of backscatter and attenuation caused by the heterogeneity.

### Breast Phantom

In order to evaluate the effect of increasing the expander volume as well as the presence of the heterogeneity in a clinical treatment planning, a sliced breast phantom containing the magnetic disk, described previously as heterogeneity Type III, was attached to the thorax of an Alderson Rando anthropomorphic phantom [21].

Some studies have already been done with breast phantoms [22]–[26], the majority interested in reproduce breast tissue properties related to diagnosis. For this work, an agar phantom was developed to reproduce all the aspects of interest like shape, density, volume and easy insertion of the magnetic disk inside the phantom. The agar phantom consists of 15 g of agar per liter of water and has density of 1.06 g/cm^3^.

**Figure 6:**
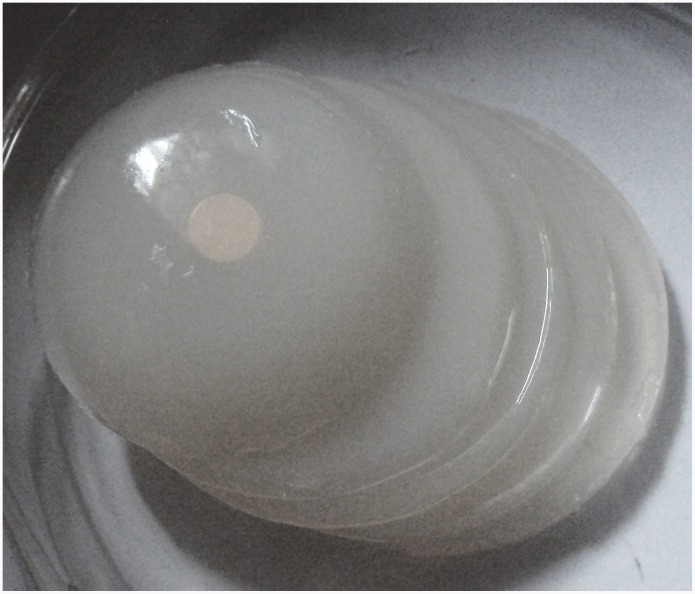
Breast phantom composed by five layers with the magnetic disk fixed at the first layer.

The sliced breast phantoms were manufactured in order to reproduce the planning of a typical radiotherapy breast treatment of 25 fractions. Five different phantom slices were produced, the first slice containing the magnetic disk fixed at 1.5 cm from the top surface (Fig. 6), representing the expander smallest volume. Others four slices, each one with 100 ml, were constructed allowing gradual volumes expansions. The last four volumes were achieved stacking the slices of 100 ml, producing partial volumes of 250, 350, 450 and 550 ml.

### Evaluation of a Clinical Treatment Planning

A set of five computer tomography (CT) images of the phantom were performed on Brilliance Big Bore CT scanner (Philips Medical System).

A post-mastectomy radiotherapy treatment was planned to a prescribed dose of 5000 cGy with a pair of opposite tangent 6 MV photon beam of 12×15 cm^2^ field size defined at the entrance surface. As skin and pectoral muscle that should be treated are in the surface of the breast in reconstruction, a bolus with 1 cm thickness was added in order to move the build-up region for this area.

Two different approaches were calculated by Eclipse™/Varian [27] treatment planning system. The first one considered a homogeneous breast, assigning the density of 1 g/cm^3^ (water), independently of the Hounsfield Unit (HU), and the other regarding the magnetic heterogeneity. The magnetic heterogeneity originated artifacts in the CT image and consequently uncompleted HU data. Then, in order to overcome this problem, an automatic identification of CT numbers belonging to the artifact was conducted but the CT number attributed to the heterogeneity was above the saturation limit of the system. Therefore, the heterogeneity was manually inserted in each slice where it appears, for the whole set of images. Due to the limitation of the system in creating a structure smaller than the pixel image (∼0.7 mm), just the major part (magnetic part) of the disk was taken in account, since the metal casing where it is involved is in the range of micrometer (∼30 µm). At this point, the Pencil Beam Convolution (Version 8.6.15) Algorithm was applied, and the density of the magnetic heterogeneity was defined as being 5 g/cm^3^, the limit value allowed by the system.

To evaluate the clinical parameters, a map of isodose curves was constructed for each volume, one considering heterogeneity correction and another disconsidering it. The two approaches and the five volumes were compared by these maps in order to assess alterations in the dose distributions due to the heterogeneity or to expander volume changes.

## Results

### Monte Carlo Simulations

The results comparing the effect produced by the bone ribs are presented on Table 1. Values of calculated absorbed energy and the respective composition simulated for chest wall structure in each case are presented.

**Table 1:** Deposited energy for chest wall irradiation with different compositions.

CHEST WALL	DEPOSITED ENERGY (MeV)	
Composition and Thickness	With Heterogeneity	Without Heterogeneity
1.0 cm of Muscle Tissue	2.598×10^−2^	2.604×10^−2^
0.4 cm of Muscle Tissue +0.6 cm of Bone Tissue	3.2574×10^−2^	3.2663×10^−2^
1.0 cm of Bone Tissue	3.6159×10^−2^	3.598×10^−2^

Although the energy deposited in structures of different compositions presents different values, as expected since they have different densities and attenuation coefficients, the difference between values of absorbed energy for the structure in the presence and absence of magnetic material presents percentage variation less than 0.5%. It is important to highlight that this result is achieved even for the limit case considered, where the complete structure was represented as being constituted uniquely by bone. After this result, the ribs bones were removed from the irradiation geometry and the chest wall was considered as composed by only muscle tissue.

**Figure 7:**
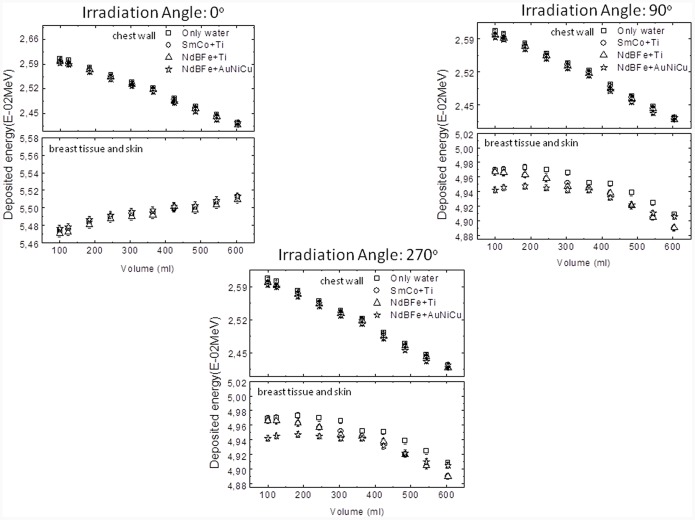
Deposited energy curves calculated with MCNP for the three heterogeneity types studied on the structures representing chest wall and breast tissue and skin (A) 0°, (B) 90° and (C) for 270° irradiation, respectively.

The evaluation of the energy deposited in the chest wall and breast tissue/skin for the three types of magnetic heterogeneities as function of the expander volume is presented in Fig. 7, to 0° and opposed beams of 90° and 270°, respectively. Irradiation simulation in the absence of any type of heterogeneity (only water) is represented by squares, heterogeneity type I (SmCo+ Ti) by circles, type II (NdBFe+Ti) by triangles and type III (NdBFe+AuNiCu) by stars.

As can be observed, variations in the expandeŕs volume leads to a gradual decrease of the deposited energy in the structure that is located under it, except to breast tissue/skin for 0° irradiation where the deposited energy increases steadily due to the backscattering. This effect is clearly caused by the increase of the water layer thickness, which attenuates photons from the incident beam. With respect to the presence of the heterogeneity, the energy deposited in the chest wall doesńt change significantly for any of the three magnetic heterogeneities types studied.

At the larger volume studied, differences in the deposited energy at breast tissue and skin structure is around 7% (0°) and 1% (90°) in comparison with the first studied volume. The deposited energy calculated for the structure that represents the chest wall does not seem to be affected significantly by the change in the expander volume. No significant difference was observed for both irradiation angles.

**Figure 8:**
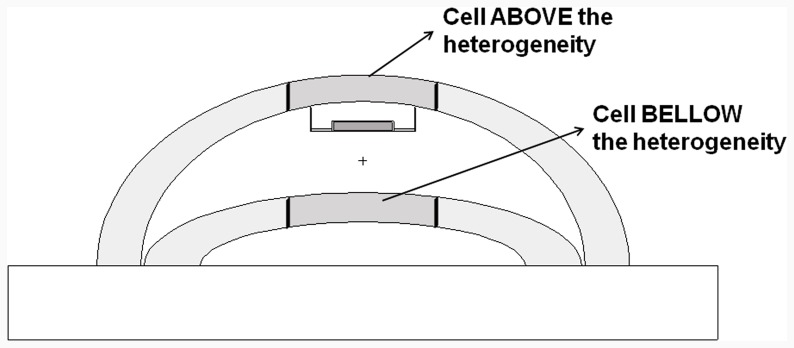
Breast phantom where structures constructed above and below the heterogeneity can be visualized.

The second step was to perform the deposited energy calculations only for structurés parts (cells) that lie just above and below the artifact to verify the existence of hot or cold spots (Fig. 8).

In order to quantify these effects a Heterogeneity Factor (HF) was defined as the ratio between the values of deposited energy with and without the presence of the artifact. Three sample volumes were chosen in order to assess each effect, for irradiations at 0°. For the evaluation of attenuation, small volumes were chosen due to the disk proximity to the structure below it, and for backscattering evaluation three arbitrary volumes were tested: 100 ml, 364 ml and 600 ml. To evaluate backscattering and attenuation effects for opposed beams irradiation, five sample volumes were used. The first three volumes (100 ml, 124 ml and 184 ml), an intermediary (364 ml) and the larger one (600 ml) were studied. Tables 2 and 3 show the HF for these volumes.

**Table 2:** HF values for irradiation at 0° for the three types of heterogeneity studied.

0° irradiation			
Bellow the Heterogeneity			
HF			
Volume	Type I	Type II	Type III
100 ml	0.95	0.97	0.89
124 ml	0.96	0.97	0.90
184 ml	0.97	0.98	0.93
**Above the Heterogeneity**			
**HF**			
Volume	Type I	Type II	Type III
100 ml	1.00	1.00	1.01
364 ml	1.00	1.01	1.00
600 ml	1.00	0.95	1.02

**Table 3:** HF values for opposed pair irradiation for the three types of heterogeneity studied.

Opposed pair: 90°+270° irradiation			
Bellow the Heterogeneity			
HF			
Volume	Type I	Type II	Type III
100 ml	1.01	1.01	1.00
124 ml	1.01	1.00	1.00
184 ml	1.00	1.00	0.99
364 ml	1.00	1.00	0.99
600 ml	1.00	1.00	0.99
**Above the Heterogeneity**			
**HF**			
Volume	Type I	Type II	Type III
100 ml	1.00	1.00	0.99
364 ml	1.00	1.00	1.00
600 ml	1.00	1.00	1.00
364 ml	1.00	1.00	1.00
600 ml	1.00	1.00	1.00

It can be observed that the attenuation effect is inversely linked with the expander volume for the irradiation at 0° and is clearly caused by photons that are attenuated by the disk high density material and don’t reach the structure as expected. Therefore this effect is valid only for volumes where the disk is close to the structure; otherwise it couldn’t be associated to the presence of the magnetic disk but to the rise in the water layer thickness. A maximum was observed for the heterogeneity Type III on the first volume studied (HF = 0.89), and even for the last volume studied (184 ml) the attenuation is significant (HF = 0.93). The smaller HF values obtained for heterogeneity type III, in both 0° and opposed beam irradiation, is probably due to the fact that it has materials with higher atomic number on its surface (Z_gold_ = 79, Z_copper_ = 28 and Z_nickel_ = 29) compared with the others (Z_titanium_ = 22), despite to be geometric smaller. Backscattering effects are insignificant (none HF >1.02) for the volumes studied, with respect to the three heterogeneity types investigated for the two irradiation positions.

### Evaluation of a Treatment Planning

The maps of isodose curves with and without heterogeneity correction, for each volume, were compared.

**Figure 9:**
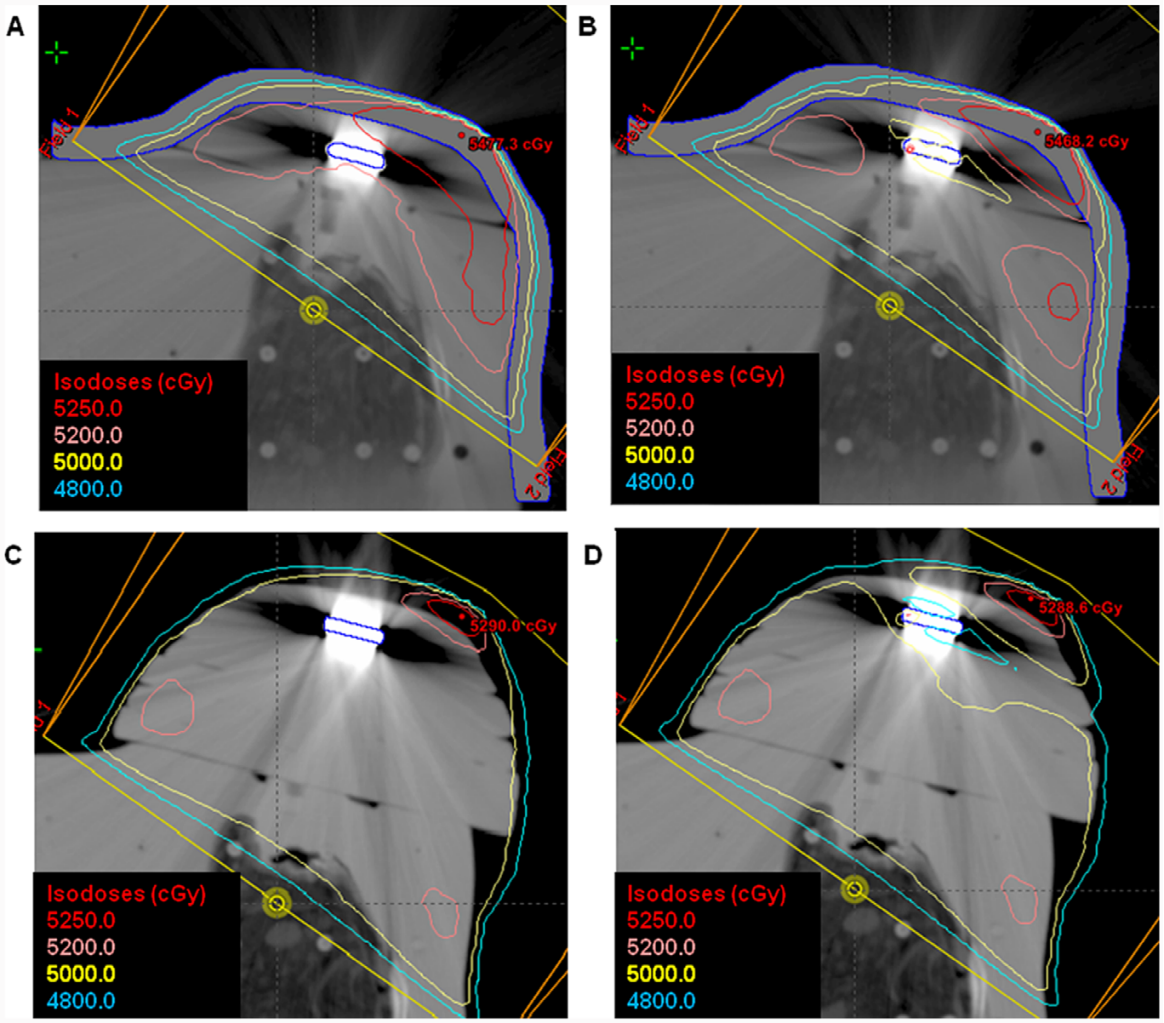
Treatment Planning isodose Curves for the two extremes volumes considered with and without heterogeneity correction algorithm applied. The curve of 5000cGy is the prescribed curve. (A) Planning Isodose curves for the first volume without heterogeneity correction and (B) with correction; (C) Planning Isodose curves for the last volume without heterogeneity correction and (D) with correction.

An underdose region on both sides of the magnetic disc appears when the correction for heterogeneity was applied and increases with the volume. This effect can be seen in Fig. 9 which shows the planning for the two extremes volumes with and without the heterogeneity correction algorithm. This hall of underdose is clearly caused by the angulation of the magnetic disc with respect to the incidence angle of the radiation beam. For larger volumes the disc becomes parallel to the beam, increasing the thickness of high density material, defining the path of beam entry and exit in the breast phantom.

In Despite of the observation of different patterns in the isodose curves to all studied volumes when the correction for heterogeneity is applied, numerically none of these alterations was higher than 5% of the prescribed dose and are, in most of cases, limited to the expander volume, not reaching significantly the treatment volume.

Hot spots were not found in the treatment region. But is important to mention that the treatment planning system does not consider electrons transport in the calculations, which could be responsible for this type of effect.

Even with the limitation of the treatment planning system in reproduce the geometry and real density of the magnetic disc, and the absence of electron transport calculations, the results found by this approach confirm what was found with Monte Carlo simulations, e.g, that the presence of the heterogeneity didn’t alter the deposited dose (energy) on treatment structures when a traditional pair of opposite tangent 6 MV photon beam is used.

### Conclusion

The influence of the magnetic heterogeneity inside tissue expanders was studied at a first approach using Monte Carlo simulations. The study was performed through the evaluation of the deposited energy values in structures which have clinical importance for the treatment considering three types of magnetic disk and irradiation with a 6 MV photon beam in three different angles. Expansion of the prosthesis was also taken into account.

Underdosage of 7% was found for the larger volume of breast implant, in the case of frontal field irradiation for the chest wall, indicating that the change in breast expander volume alters the deposited energy to this angle of beam.

Variations in the energy deposited due to the presence of the heterogeneity in the radiation field were observed mainly for irradiation at 0°, as could be also seen in the literature. And the alterations show great values of attenuation mostly for the tissue expander manufactured by SILIMED, never studied before. These large differences are presumably due to the higher atomic numbers of materials that compose the magnetic disk. Insignificant variations were observed for irradiation with opposed pair beams.

It should be remembered that direct irradiations with a beam at 0° are not commonly used at clinical practice in order to avoid great lung doses, and it has been considered at this work in the Monte Carlo simulations with the intention of assessing the results found in the literature. But it is also worth remembering that anatomical expanders have the magnetic valve positioned in many different places, and depending where it is positioned the incident radiation beam could have an angle of interaction with the magnetic disk close to the presented by the 0° angle of irradiation showed by this work.

To contrary to the literature values for attenuation and dose enhancement did not match exactly with the results found at this work, it has to emphasize that this work was concerned only with deposited energy on structures with clinical importance, regardless of the dose distribution inside the expander aqueous volume. Most of differences found by other works were at points close to the valve, which have probable localization inside the aqueous volume of the expander.

Despite the fact that little differences were found for the irradiation with a opposed pair of beams, the results suggest that patients implanted with heterogeneity of type III could have a greater possibility of failure on delivered dose, mainly below the heterogeneity (inside the breast) during the irradiation treatment, since the treatment can be executed with a beam positioned in angles between the extremes angles considered in this work.

It should be remembered that direct irradiations with a beam at 0° are not commonly used at clinical practice in order to avoid great lung doses, and it has been considered at this work with the intention of assessing the results found in the literature.

Since deposited energy is not directly related to clinic use, and with the aim of verifying if the heterogeneity alters the dose distribution, a conventional breast treatment planning was performed for five different expander volumes using CT images acquired from a anatomic breast phantom containing the magnetic disk type (heterogeneity Type III), which lead major changes in the study performed by Monte Carlo simulations.

Isodose curves show an underdose area at both sides of the magnetic disk when the correction for heterogeneities is applied. The results presented by Chatzigiannis *et al.* [15] agree in the location of underdose areas although they described greater values of underdose (6–13%), compared with what was found here (below 5%). Thompson and Morgan [16] also described affects of attenuation in the same region (in order of 23%) but to smaller distance from the artifact, inside the expander tissue. Both studies consider the real density of heterogeneity (to other type of tissue expander), what was not possible with the planning system used for this work for the type III of expander. It is important to take into account the heterogeneity position with respect to the incidence angle of radiation beam; the two studies previously mentioned, as well as this work, found high underdose effects for angles of incident irradiation beam parallel to the heterogeneity position. This effect is easily explained by the enhancement of high density material thickness that the beam crosses at this situation. Overdose effects were not observed.

Many difficulties were found in the planning stage, as the presence of artifacts in the image modify the CT numbers of the structures and alters the real information contained in the image, leading to the necessity of redesign the heterogeneity limits in each slice of the CT set images, and also the limitation of the system in defining the real density of the heterogeneity and ignore the generations of electrons.

The results found in this study shows little influence of the presence of the magnetic disk for radiotherapy breast treatment and suggests that the high rate of complications and reconstruction failure should be related to other relevant parameters, like the biological aspects related to the irradiated tissues during the reconstruction process, as discussed by Ozden et al [28].

No significant differences were found for the presence of the heterogeneity during the treatment planning of irradiation with an opposed pair of beams. Most of differences found by other works were at points close to the magnetic disk, which have probable localization inside the aqueous volume of the expander, not being important for the treatment because it is not a structure to be treated.
